# Análise das condições operacionais para conservação de imunobiológicos nas salas de vacinação do Brasil: estudo misto

**DOI:** 10.1590/0102-311XPT014924

**Published:** 2024-08-23

**Authors:** Gabriela Gonçalves Amaral, Luísa Gomes de Sousa, Samuel Pereira da Silva, Ana Luíza Karter, Brener Santos Silva, Fabiana Costa Machado Zacharias, Tatiele Estefâni Schonholzer, Ana Catarina de Melo Araújo, Valéria Conceição de Oliveira, Ione Carvalho Pinto

**Affiliations:** 1 Escola de Enfermagem de Ribeirão Preto, Universidade de São Paulo, Ribeirão Preto, Brasil.; 2 Universidade do Estado de Minas Gerais, Divinópolis, Brasil.; 3 Universidade Federal de São João del-Rei, Divinópolis, Brasil.; 4 Universidade Federal do Paraná, Curitiba, Brasil.; 5 Departamento do Programa Nacional de Imunizações, Ministério da Saúde, Brasília, Brasil.

**Keywords:** Programas de Imunização, Rede de Frio, Vacinação, Enfermagem, Immunization Programs, Cold Chains, Vaccination, Nursing, Programas de Inmunización, Cadena Fría, Vacunación, Enfermería

## Abstract

Este estudo objetivou analisar as condições operacionais para conservação de imunobiológicos no Brasil. Trata-se de um estudo de método misto com delineamento explanatório sequencial, desenvolvido nas salas de vacinação de distintas regiões brasileiras entre 2021 e 2022. Na etapa quantitativa, desenvolveu-se um estudo transversal descritivo, com a aplicação da *Escala de Avaliação da Conservação de Imunobiológicos* aos profissionais de enfermagem. Os dados foram analisados por meio de estatística descritiva. Já na etapa qualitativa, desenvolveu-se um estudo descritivo-exploratório nas instâncias da cadeia de frio, com os respectivos responsáveis técnicos e profissionais de enfermagem. Analisou-se as entrevistas por meio da Análise de Conteúdo na Modalidade Temática. Os dados foram combinados mediante conexão, com elaboração de *joint-displays* e metainferências. Foram analisadas 280 salas, em que grande parte: era de uso exclusivo (79,6%); utilizava caixas de poliuretano (77,8%); e mantinha seus equipamentos distantes da incidência de luz solar/fontes de calor (73,5%). Apenas 27,5% dispunham de baterias/geradores e 26,5% de outros instrumentos de medição de temperatura. Sessenta por cento detinham câmaras refrigeradas e 67,6% ambientes climatizados. Revelaram-se fragilidades associadas a condições geográficas, infraestrutura, insumos materiais, recursos humanos e financeiros, organização e gestão do trabalho, rotatividade e capacitação. Os achados possibilitaram conhecer a pluralidade da cadeia de frio brasileira e permitiram a identificação de potencialidades e fragilidades na conservação de imunobiológicos relacionadas às estruturas e aos processos de trabalho que requerem aprimoramento.

## Abstract (en)

This study aimed to analyze the operational conditions to preserve immunobiological products in Brazil. This mixed-method study with a sequential explanatory design was developed in vaccination rooms in several Brazilian regions from 2021 to 2022. Its quantitative stage developed a descriptive cross-sectional study by applying the *Immunobiological Conservation Assessment Scale* to nursing professionals. Data were analyzed by descriptive statistics. Its qualitative stage developed a descriptive-exploratory study in cold chain instances with the respective technical managers and nursing professionals. The interviews were evaluated by thematic content analysis. The data were combined by connection, and joint-displays and meta-inferences were elaborated. Overall, 280 rooms were analyzed. Most were for exclusive use (79.6%), had polyurethane boxes (77.8%), and kept their equipment away from sunlight/heat (73.5%). Only 27.5% had batteries/generators and 26.5% had other temperature measuring instruments. In total, 60% had refrigerated rooms and 67.6%, air-conditioned rooms. This study found weaknesses associated with geographical conditions, infrastructure, material inputs, human and financial resources, work organization and management, turnover, and training. These findings showed the plurality of the Brazilian cold chain and identified the potentialities and weaknesses related to the structures and work processes in preserving immunobiological products, which require improvement.

## Resumen

Este estudio tuvo como objetivo analizar las condiciones operativas para la conservación de inmunobiológicos en Brasil. Estudio de método mixto con diseño explicativo secuencial, desarrollado en las salas de vacunación de las distintas regiones brasileñas, entre 2021 y 2022. En la etapa cuantitativa se desarrolló un estudio transversal descriptivo, con la aplicación de la *Escala de Evaluación de la Conservación de Inmunobiológicos* a los profesionales de enfermería. Los datos fueron analizados mediante estadística descriptiva. En la etapa cualitativa se desarrolló un estudio descriptivo-exploratorio en las instancias de la cadena de frío, con los respectivos responsables técnicos y profesionales de enfermería. Las entrevistas se analizaron por medio del Análisis de Contenido en la Modalidad Temática. Los datos fueron combinados mediante conexión, con elaboración de *joint-displays* y metainferencias. Se analizaron 280 salas. Gran parte era de uso exclusivo (79,6%); utilizaba cajas de poliuretano (77,8%) y mantenía sus equipos alejados de la incidencia de la luz solar/fuentes de calor (73,5%). Solo el 27,5% disponía de baterías/generadores y el 26,5% de otros instrumentos de medición de temperatura. El 60% tenía cámaras refrigeradas y el 67,6% ambientes climatizados. Se revelaron debilidades asociadas a las condiciones geográficas, la infraestructura, los insumos de materiales, los recursos humanos y financieros, la organización y gestión del trabajo, la rotación y la capacitación. Los hallazgos posibilitaron conocer la pluralidad de la cadena de frío brasileña y permitieron la identificación de potencialidades y debilidades de la conservación de inmunobiológicos relacionadas con las estructuras y los procesos de trabajo que requieren mejoras.

## Introdução

No Brasil, as atividades de vacinação se constituem como uma das ações prioritárias das unidades de (APS) em outros níveis de atenção à saúde e em menor proporção, como hospitais e unidades de pronto atendimento. O Programa Nacional de Imunizações (PNI) dispõe de políticas, normas e diretrizes que envolvem os processos relacionados à vacinação, desde a aquisição dos imunobiológicos até a administração desses nas salas de vacinação do país ^1^. É nesses locais que todo o trabalho implementado e coordenado pelas esferas de gestão do PNI se concretiza, viabilizando a administração dos imunobiológicos de acordo com as normatizações estabelecidas.

O processo de vacinação não engloba apenas o alcance da cobertura vacinal, mas também as condições próprias de preparo, administração e armazenamento dos imunobiológicos ^2^ ^,^ ^3^. Para tanto, inúmeros aspectos das salas de vacinação requerem normatizações, visando a adequada conservação desses produtos.

Nesse sentido, destaca-se a cadeia de frio, definida pelo trajeto percorrido pelos imunobiológicos desde o laboratório produtor até sua administração no usuário. Essa se estratifica em instâncias: nacional, estaduais, regionais, municipais e locais, as últimas caracterizadas pelas salas de vacinação ^1^ ^,^ ^4^. Logo, esse processo logístico demanda sistematizações como recebimento, armazenamento, distribuição e transporte dos imunobiológicos visando a manutenção da temperatura adequada dos produtos, haja vista suas termolabilidades e, consequentemente, a importante seguridade de suas imunogenicidade ^1^ ^,^ ^5^.

Embora haja orientações estipuladas pelo PNI brasileiro, os estados e municípios têm autonomia quanto ao planejamento das ações de vacinação, além de estruturação e organização das salas de vacinação ^1^ ^,^ ^6^. Assim, levando em consideração a grande extensão territorial do Brasil, faz-se necessário compreender as particularidades e dificuldades das salas de vacinação no que tange à conservação de imunobiológicos nas distintas regiões brasileiras.

Os estudos anteriores realizados nessa perspectiva utilizaram o Programa de Avaliação do Instrumento de Supervisão em Sala de Vacinação (PAISSV), criado em 2003. Esse instrumento está desatualizado em virtude dos avanços na conservação de imunobiológicos. Nesse ínterim, este estudo avança ao utilizar uma escala validada que auxilia o processo de supervisão da cadeia de frio nas salas de vacinação, identificando a conformidade com as normas estabelecidas pelo PNI e aspectos que necessitam de aprimoramento. Ademais, o estudo incorpora a abordagem qualitativa para uma compreensão mais aprofundada da cadeia de frio, explorando como as particularidades do contexto brasileiro podem influenciar esse processo.

Essa identificação auxilia os gestores na otimização dessa cadeia, promovendo constantes melhorias para alcançar um padrão cada vez mais elevado de segurança e efetividade na conservação de imunobiológicos. Nesse contexto, este estudo objetivou analisar as condições operacionais para conservação de imunobiológicos no Brasil.

## Métodos

Trata-se de um estudo de método misto, com delineamento explanatório sequencial (QUAN → qual) ^7^ e desenvolvido entre 2021 e 2022 nas salas de vacinação das unidades de APS de 80 municípios, distribuídos nas distintas regiões brasileiras.

A etapa quantitativa se tratou de um estudo transversal descritivo para análise das condições operacionais das salas de vacinação em relação à conservação de imunobiológicos. Na etapa qualitativa, desenvolveu-se um estudo descritivo-exploratório a fim de compreender a conservação de imunobiológicos no contexto brasileiro.

Para a etapa quantitativa, selecionou-se os estados, municípios e salas de vacinação por amostragem probabilística, haja vista a necessidade de obter representatividade e conferir validade interna e externa ao estudo. Para tanto, foram utilizadas as informações dos municípios e estados acerca da população total e da taxa de cobertura da vacina pentavalente (vacina contra difteria, tétano, coqueluche, hepatite B e *Haemophilus influenzae* tipo b - DTPw-HB/Hib), ambas referentes a 2019 para minimizar a influência da pandemia de COVID-19 ^8^ ^,^ ^9^. O procedimento de amostragem ^10^ foi realizado em três estágios (estado, município, sala de vacinação):

(a) Estados: realizou-se uma amostragem por conglomerados e pelo método de probabilidade proporcional ao tamanho, por meio da informação da população total e localização de cada estado, exceto o Distrito Federal, que foi considerado um conglomerado. O cálculo amostral gerou um número de oito conglomerados (estados), os quais foram sorteados dentro das regiões brasileiras (Norte: Amazonas; Nordeste: Ceará, Pernambuco e Bahia; Centro-oeste: Goiás; Sudeste: Rio de Janeiro e São Paulo; e Sul: Santa Catarina);

(b) Municípios: realizou-se um procedimento de amostragem aleatória simples para a seleção dos municípios dentro dos estados sorteados, gerando uma amostra de 10 por estado. Considerou-se para a taxa de cobertura vacinal (DTPw-HB/Hib) os parâmetros de nível de significância de 5% (α = 0.05) e uma diferença de 10% entre o valor estimado e o verdadeiro (d = 0.10) ^11^. Para fins de representatividade, incluiu-se três estratos segundo o porte populacional (sete com até 20.000 habitantes; dois entre 20.001 e 100 mil habitantes; e um acima de 100 mil habitantes) ^11^, totalizando 80 municípios. Posteriormente, procedeu-se sorteios dos municípios dentro dos estados e estratos populacional;

(c) Salas de vacinação: após aceitar a participação, foi fornecido pelos municípios o total de salas de vacinação em seu território, contabilizando-se 475. Assim, uma amostragem aleatória simples se procedeu para a seleção dessas dentro de cada município, obtendo uma amostra de 227 salas de vacinação selecionadas mediante sorteio.

O sorteio dos estados e municípios foi realizado de modo independente por um estatístico, sem vinculação ao projeto ou quaisquer interferências dos pesquisadores; já o sorteio das salas coube à pesquisadora principal que, de posse da listagem delas, efetuava-o utilizando um software para amostragem. Os estados, municípios e salas, previamente sorteados, foram contatados e convidados para participar da pesquisa e, em caso de rejeição, realizava-se um novo sorteio, respeitando as informações da região, estado e porte populacional.

Incluiu-se as salas de vacinação urbanas e rurais, da rede pública vinculadas às unidades de APS. Foram excluídas aquelas: destinadas apenas como ponto de apoio para as equipes de saúde das zonas rurais pela ausência de estrutura física; localizadas em hospitais e/ou unidades de pronto atendimento; e inativadas ou ativas apenas para o período da campanha de vacinação contra a COVID-19.

Para seguimento da etapa qualitativa, foram convidados a participar os responsáveis técnicos em imunização (RTI) pelas instâncias da cadeia de frio estadual (RTIE), regional (RTIR), municipal (RTIM) e profissionais da equipe de enfermagem atuantes nas respectivas salas. Foram incluídos todos aqueles com seis meses ou mais de atuação em atividades de vacinação, dado a experiência necessária à realização das atividades relacionadas à conservação de imunobiológicos.

Utilizou-se a *Escala de Avaliação da Conservação de Imunobiológicos* (EACI) ^12^, validada para o contexto brasileiro e cuja finalidade é mensurar a manutenção da cadeia de frio de imunobiológicos nas salas de vacinação. A escala é composta por 12 itens com quatro opções de respostas (nunca, quase nunca, quase sempre, e sempre), que permitem analisar o contexto em que as salas estão inseridas e norteiam a implementação de intervenções para possíveis melhorias ^12^.

Embora tenha sido utilizada uma escala validada, foi realizado um estudo piloto em um município distinto do cenário do estudo a fim de se verificar a compreensibilidade do instrumento. Ele atestou a compreensão dos itens da EACI, não sendo necessária qualquer adaptação.

A aplicação da escala foi realizada com os profissionais atuantes nas respectivas salas de vacinação e de maneira individualizada e sistematizada, por meio de entrevista telefônica ou envio de um *link* para acesso, por aplicativo de comunicação, à escolha do participante. Os profissionais foram convidados e informados quanto à livre participação. Após aceite, foi encaminhado (via e-mail ou pelo aplicativo de comunicação) um *link* para acesso ao Termo de Consentimento Livre e Esclarecido, formalizando sua participação no estudo.

Para a etapa qualitativa, foram realizadas entrevistas individuais, por meio de plataforma *online*, com roteiro semiestruturado baseado em questões norteadoras que abordaram fatores relativos à conservação de imunobiológicos. Elaborou-se um roteiro para os responsáveis técnicos de cada instância da cadeia de frio (RTIE, RTIR, RTIM), e um para a equipe de enfermagem, haja vista as diferentes particularidades e responsabilidades de cada uma dessas (Material Suplementar; https://cadernos.ensp.fiocruz.br/static//arquivo/suppl-e00014924_3561.pdf).

As entrevistas foram gravadas em arquivo áudio digital e, posteriormente, transcritas literalmente, sendo preservada a fidedignidade das informações. O anonimato dos participantes foi garantido por meio da denominação profissional (RTIE; RTIR; RTIM; enfermeiro = ENF/técnico de enfermagem = TEC), juntamente com numeração sequencial e identificação da respectiva região brasileira (p.ex.: RTIE1-Norte; RTIR1-Sul; RTIM1-Centro-oeste; ENF1-Nordeste; TEC1-Sul).

Os dados quantitativos foram processados e analisados mediante estatística descritiva e apresentados em frequência absoluta e relativa. A análise foi implementada pelo software SPSS, versão 21.0 (https://www.ibm.com/). Os dados qualitativos foram analisados à luz da Análise de Conteúdo na Modalidade Temática ^13^, caracterizada pela análise dos significados do discurso. Posteriormente, realizou-se codificação do material empírico, identificando e selecionando os elementos que compuseram as categorias temáticas.

Após as análises, os dados qualitativos e quantitativos foram combinados mediante conexão por meio da elaboração de *joint-displays* ^14^, visando a busca por convergências e divergências apresentadas por meio de metainferências. Elas traduziram as conclusões geradas da combinação de evidências quantitativas e qualitativas, proporcionando melhor entendimento da temática em estudos mistos ^7^ ^,^ ^14^.

O estudo recebeu aprovação dos Comitês de Ética em Pesquisa da Escola de Enfermagem de Ribeirão Preto da Universidade de São Paulo (parecer nº 4.610.079); da Universidade Federal de São João del-Rei (parecer nº 4.747.228); e da Comissão Nacional de Ética em Pesquisa (parecer nº 4.812.197).

## Resultados

Foram analisadas 280 salas de vacinação nas cinco regiões brasileiras. Dessas, 127 (45,3%) estavam inseridas nas unidades de APS do tipo Estratégia Saúde da Família, modelo preferencial para organização da APS; e 219 (78,3%) se localizam na zona urbana. Na maioria das salas, o profissional enfermeiro realizava vacinação (n = 203; 72,5%) e não havia escala de trabalho (n = 185; 66,1%) (Tabela 1).

**Tabela 1:** Características das salas de vacinação segundo as regiões brasileiras, 2022 (n = 280).

Características das salas de vacinação	Regiões [n (%)]			
	Norte	Nordeste	Centro-oeste	Sudeste	Sul	Total
Local de inserção						
Unidades de APS - tradicional	19 (6,8)	81 (28,9)	16 (5,7)	16 (5,7)	7 (2,5)	139 (49,7)
Unidades de APS - ESF	8 (2,9)	62 (22,1)	14 (5,0)	34 (12,1)	9 (3,2)	127 (45,3)
Instância municipal ^*^	2 (0,7)	4 (1,4)	-	6 (2,1)	2 (0,7)	14 (5,0)
Localidade						
Zona urbana	22 (7,9)	99 (35,4)	30 (10,7)	51 (18,2)	17 (6,1)	219 (78,3)
Zona rural	7 (2,5)	48 (17,1)	-	5 (1,8)	1 (0,4)	61 (21,7)
Realização de vacinação pelo enfermeiro						
Sim	20 (7,1)	102 (36,4)	21 (7,5)	54 (19,3)	6 (2,1)	203 (72,5)
Não	9 (3,2)	45 (16,1)	9 (3,2)	2 (0,7)	12 (4,3)	77 (27,5)
Escala de trabalho						
Sim	15 (5,4)	37 (13,2)	11 (3,9)	28 (10,0)	4 (1,4)	95 (33,9)
Não	14 (5,0)	110 (39,3)	19 (6,8)	28 (10,0)	14 (5,0)	185 (66,1)

APS: atenção primária à saúde; ESF: Estratégia Saúde da Família.

* Instância municipal da cadeia de frio.

Evidenciou-se que grande parte das salas eram de uso exclusivo para as atividades de vacinação (n = 223; 79,6%); faziam uso de caixas térmicas de poliuretano na rotina diária das atividades (n = 218; 77,8%) e mantinham seus equipamentos de refrigeração distantes da incidência de luz solar ou de outras fontes de calor (n = 206; 73,5%). Contudo, em apenas parte delas era realizada manutenção preventiva desses equipamentos (n = 143; 51%). Acerca da substituição das bobinas de gelo reutilizáveis quando vencidas ou danificadas, pouco mais da metade relatou sempre as substituírem (n = 163; 58,2%) (Tabela 2).

**Tabela 2:** Características das salas de vacinação em relação à conservação de imunobiológicos segundo as regiões brasileiras, 2022 (n = 280).

Variáveis/Regiões	Nunca n (%)	Quase nunca n (%)	Quase sempre n (%)	Sempre n (%)
Sala de vacinação exclusiva para esse fim				
Norte	2 (0,7)	1 (0,4)	2 (0,7)	24 (8,6)
Nordeste	6 (2,1)	7 (2,5)	16 (5,7)	118 (42,1)
Centro-oeste	-	1 (0,4)	4 (1,4)	25 (8,9)
Sudeste	5 (1,8)	3 (1,1)	9 (3,2)	39 (13,9)
Sul	1 (0,4)	-	-	17 (6,1)
Total	14 (5,0)	12 (4,4)	31 (11,0)	223 (79,6)
Sala de vacinação equipada com câmara refrigerada para o armazenamento de imunobiológicos				
Norte	13 (4,6)	-	-	16 (5,7)
Nordeste	77 (27,5)	-	-	70 (25,0)
Centro-oeste	4 (1,4)	-	-	26 (9,3)
Sudeste	17 (6,1)	-	-	39 (13,9)
Sul	1 (0,4)	-	-	17 (6,1)
Total	112 (40,0)	-	-	168 (60,0)
Sala de vacinação climatizada (entre +18ºC e +20ºC)				
Norte	2 (0,7)	2 (0,7)	3 (1,1)	22 (7,9)
Nordeste	28 (10,0)	3 (1,1)	24 (8,6)	92 (32,9)
Centro-oeste	1 (0,4)	1 (0,4)	4 (1,4)	24 (8,6)
Sudeste	6 (2,1)	4 (1,4)	8 (2,9)	38 (13,6)
Sul	-	-	5 (1,8)	13 (4,6)
Total	37 (13,2)	10 (3,6)	44 (15,7)	189 (67,5)
Presença de outros instrumentos de medição de temperatura além do termômetro de momento, de máxima e de mínima				
Norte	10 (3,6)	3 (1,1)	4 (1,4)	12 (4,3)
Nordeste	107 (38,2)	5 (1,8)	9 (3,2)	26 (9,3)
Centro-oeste	16 (5,7)	-	2 (0,7)	12 (4,3)
Sudeste	34 (12,1)	4 (1,4)	1 (0,4)	17 (6,1)
Sul	10 (3,6)	-	1 (0,4)	7 (2,5)
Total	177 (63,2)	12 (4,3)	17 (6,1)	74 (26,4)
Incidência de luz solar/calor nos equipamentos de refrigeração				
Norte	27 (9,6)	1 (0,4)	-	1 (0,4)
Nordeste	94 (33,6)	23 (8,2)	18 (6,4)	12 (4,3)
Centro-oeste	25 (8,9)	4 (1,4)	1 (0,4)	-
Sudeste	44 (15,7)	7 (2,5)	3 (1,1)	2 (0,7)
Sul	16 (5,7)	-	1 (0,4)	1 (0,4)
Total	206 (73,5)	35 (12,5)	23 (8,3)	16 (5,7)
Manutenção preventiva dos equipamentos de refrigeração				
Norte	-	4 (1,4)	4 (1,4)	21 (7,5)
Nordeste	12 (4,3)	25 (8,9)	43 (15,4)	67 (23,9)
Centro-oeste	1 (0,4)	6 (2,1)	5 (1,8)	18 (6,4)
Sudeste	11 (3,9)	5 (1,8)	10 (3,6)	30 (10,7)
Sul	-	1 (0,4)	10 (3,6)	7 (2,5)
Total	24 (8,6)	41 (14,6)	72 (25,8)	143 (51,0)
Disponibilidade de baterias ou geradores para casos de falhas na rede elétrica				
Norte	15 (5,4)	4 (1,4)	2 (0,7)	8 (2,9)
Nordeste	104 (37,1)	3 (1,1)	7 (2,5)	33 (11,8)
Centro-oeste	15 (5,4)	-	1 (0,4)	14 (5,0)
Sudeste	33 (11,8)	3 (1,1)	3 (1,1)	17 (6,1)
Sul	9 (3,2)	1 (0,4)	3 (1,1)	5 (1,8)
Total	176 (62,8)	11 (4,0)	16 (5,7)	77 (27,5)
Uso de caixas térmicas de poliuretano				
Norte	-	2 (0,7)	9 (3,2)	18 (6,4)
Nordeste	7 (2,5)	2 (0,7)	23 (8,2)	115 (41,1)
Centro-oeste	1 (0,4)	-	3 (1,1)	26 (9,3)
Sudeste	3 (1,1)	-	10 (3,6)	43 (15,3)
Sul	-	-	2 (0,7)	16 (5,7)
Total	11 (4,0)	4 (1,4)	47 (16,8)	218 (77,8)
Substituição das bobinas de gelo reutilizáveis vencidas				
Norte	-	1 (0,4)	7 (2,5)	21 (7,5)
Nordeste	9 (3,2)	15 (5,4)	33 (11,8)	90 (32,1)
Centro-oeste	3 (1,1)	6 (2,1)	8 (2,9)	13 (4,6)
Sudeste	5 (1,8)	10 (3,6)	10 (3,6)	31 (11,1)
Sul	5 (1,8)	-	5 (1,8)	8 (2,9)
Total	22 (7,9)	32 (11,4)	63 (22,5)	163 (58,2)
Profissional capacitado em imunização				
Norte	1 (0,4)	6 (2,1)	6 (2,1)	16 (5,7)
Nordeste	4 (1,4)	19 (6,8)	55 (19,6)	69 (24,6)
Centro-oeste	-	3 (1,1)	6 (2,1)	21 (7,5)
Sudeste	7 (2,5)	16 (5,7)	10 (3,6)	23 (8,2)
Sul	-	6 (2,1)	5 (1,8)	7 (2,5)
Total	12 (4,3)	50 (17,8)	82 (29,3)	136 (48,6)
Recebimento de informações sobre atualizações em imunização				
Norte	-	1 (0,4)	4 (1,4)	24 (8,6)
Nordeste	-	8 (2,9)	38 (13,6)	101 (36,1)
Centro-oeste	-	2 (0,7)	3 (1,1)	25 (8,9)
Sudeste	6 (2,1)	2 (0,7)	11 (3,9)	37 (13,2)
Sul	-	2 (0,7)	6 (2,1)	10 (3,6)
Total	6 (2,1)	15 (5,4)	62 (22,1)	197 (70,4)
Transporte (da instância municipal para as salas de vacinação) em carro climatizado ou com ar-condicionado ligado				
Norte	4 (1,4)	2 (0,7)	6 (2,1)	17 (6,1)
Nordeste	21 (7,5)	9 (3,2)	43 (15,4)	74 (26,4)
Centro-oeste	1 (0,4)	-	4 (1,4)	25 (8,9)
Sudeste	8 (2,9)	3 (1,1)	10 (3,6)	35 (12,5)
Sul	1 (0,4)	1 (0,4)	5 (1,8)	11 (3,9)
Total	35 (12,6)	15 (5,4)	68 (24,3)	162 (57,8)

No que tange à capacitação dos profissionais atuantes nas salas, menos da metade relatou sempre participar de capacitações em imunização (n = 136; 48,6%), entretanto, uma parcela significativa informou sempre receber informações sobre as atualizações em imunização (n = 197; 70,4%) (Tabela 2).

Em apenas 77 (27,5%) das salas se observou disponibilidade de baterias ou geradores para casos de falhas da rede elétrica; e em 74 (26,4%), a presença de outros instrumentos de medição de temperatura além do termômetro de momento, máxima e mínima. Tais itens foram menos frequentes nas salas da Região Nordeste (Tabela 2). Em oito salas (2,9%), o termômetro analógico de mercúrio/álcool ainda era utilizado (dado não apresentado em tabela).

Foram encontradas câmaras refrigeradas (n = 168; 60%) e ambientes climatizados (n = 189; 67,5%) em mais da metade das salas (Tabela 2). Evidenciou-se que tais equipamentos foram adquiridos, em sua maioria, antes de 2020 (dado não apresentado em tabela). Grande parte das salas que não dispunham de câmaras refrigeradas (n = 77; 27,5%) e com ambientes não climatizados (n = 28; 10%) se concentravam na Região Nordeste (Tabela 2).

Em parte (n = 162; 57,8%), o transporte da instância municipal para as salas sempre era realizado em carro climatizado ou com ar-condicionado ligado (Tabela 2).

Para analisar de forma mais abrangente a conservação de imunobiológicos nas salas de vacinação do país, foram realizadas 42 entrevistas com RTIE (n = 7); RTIR (n = 11); RTIM (n = 11); e profissionais atuantes nas salas (n = 13). Desses, a maioria eram mulheres (85,7%) e enfermeiras (76,1%), com vínculos contratuais efetivos (66,6%), idades entre 23 e 64 anos (mediana = 41 anos) e tempo de atuação na área variando entre um e 44 anos.

A partir das análises, emergiram-se duas categorias temáticas “Estrutura das salas de vacinação: realidades da pluralidade brasileira” e “O processo da manutenção da cadeia de frio: o cotidiano das salas de vacinação brasileiras”, que revelaram diversidades das instâncias da cadeia de frio nas distintas regiões brasileiras e repercussões na conservação de imunobiológicos.

### Estrutura das salas de vacinação: realidades da pluralidade brasileira

Entraves relacionados à estrutura física das salas podem impactar na manutenção das temperaturas e, consequentemente, na qualidade dos imunobiológicos oferecidos à população:

“*A sala de vacinação é em um ponto que eu acho que não foi muito bem pensado, porque na parte da tarde, que é o período mais quente, ela fica justamente no lado do sol, e como ela não é climatizada, para gente é uma preocupação muito grande. Ela não tem janela, mas tem a porta da sala de vacina e uma porta na lateral, aí a gente sempre vacina com as duas abertas para ter uma circulação melhor de ar* (...) *A gente se preocupa muito com a qualidade do imunobiológico, mas são questões externas que não depende da gente*” [ENF1-Nordeste].

As salas de vacinação localizadas na zona urbana geralmente apresentam melhores condições para acessibilidade. Em contraposto, aquelas localizadas em áreas rurais, por serem distantes e menos acessíveis, podem repercutir desafios logísticos para o transporte e o armazenamento dos imunobiológicos.

“*Para a gente aqui da zona urbana é muito tranquilo. Eu vejo a equipe de vacinação da zona rural sofrer com o transporte de vacinas, principalmente no período chuvoso, é bem complicado*” [ENF2-Nordeste].

“*Os carros ficam quebrados com muita facilidade porque não aguenta o rojão de todos os dias ir para a estrada de chão e às vezes quebra no percurso e tem que voltar*” [RTIM1-Nordeste].

A falta de compreensão sobre a necessidade de carro climatizado para o transporte de imunobiológicos por parte de profissionais que ocupam cargos na gestão municipal também influencia na logística da cadeia de frio:

“*Eu não tenho um carro específico da vacina e aí sempre a gente tem esse embate, porque nós, profissionais técnicos, nós sabemos da real necessidade da vacina, né? Nós sabemos das exigências a cumprir metas* (...) *Aí as pessoas que estão nos cargos que direciona os carros, eles não sabem dessa devida importância*” [RTIM2-Nordeste].

Com relação às instâncias municipais, suas estruturas físicas impactam na capacidade de armazenamento dos imunobiológicos:

“*O espaço da instância municipal começa a ficar pequeno. Principalmente com a questão da introdução das vacinas da COVID-19, a nossa capacidade acabou não ficando satisfatória para a quantidade de vacinas a receber. A instância municipal não tem um espaço só para acondicionar as vacinas, a gente tem que ter um espaço para a parte administrativa, espaço para guardar materiais que chegam, seringas, cadernetas*…” [RTIM3-Nordeste].

Desde 2017, a Agência Nacional de Vigilância Sanitária (Anvisa) brasileira instituiu a substituição do refrigerador doméstico por câmara refrigerada haja vista os benefícios para a manutenção das temperaturas, fato evidenciado pela predominância de salas munidas de tal equipamento:

“*A gente tem praticamente todas as salas de vacina com uma câmara refrigerada e não mais o refrigerador doméstico, porque a gente tem uma portaria que define que precisa ser assim* (...) *A gente sabe que ainda vai encontrar uma ou outra com refrigerador doméstico, mas a gente orienta sempre que já passe para câmara refrigerada…*” [RTIE1-Sul].

“*Eu vou lá e digo: olha, isso não pode, você está usando geladeira doméstica e desde 2017 saiu a RDC nº 197 de 2017 que diz que não pode mais armazenar essas vacinas em refrigerador doméstico, tem que ser a câmara refrigerada* (...) *O Ministério da Saúde e Anvisa deram um prazo de dois anos para isso*” [RTIE2-Nordeste].

Diante da instabilidade da rede elétrica citada nos depoimentos, torna-se relevante dispor de fontes de energia reservas em associação à elaboração de planos de contingência, principalmente para localidades de zona rural:

“*A gente tem um grande problema porque aqui falta muita energia. A unidade fica sem sinal de telefone* (...) *Só tem a casa de um vizinho que tem telefone, aí ele liga para mim ou para o técnico de enfermagem* (...) *A gente liga para o pessoal da equipe da instância municipal, o técnico de enfermagem coloca as vacinas na caixa térmica e eles vêm recolher* [ENF3-Nordeste].

### O processo da manutenção da cadeia de frio: o cotidiano das salas de vacinação brasileiras

A ambientação das bobinas de gelo reutilizáveis se configura como um procedimento essencial na mitigação de riscos potenciais de congelamento dos imunobiológicos. No dia a dia das salas, são observadas distintas variações na realização desse procedimento, as quais necessitam de revisão e reorientação:

“*Pra agilizar, um dia antes a gente pega os gelox e monta todas as caixas que a gente vai utilizar no próximo dia. No próximo dia, quando a equipe chega, elas avaliam todas as caixas. Se elas avaliarem que o gelox descongelou demais elas trocam* (...) *Foi justamente uma forma que a gente teve de agilizar o processo*” [ENF4-Sudeste].

Práticas de organização do equipamento de armazenamento que visem evitar a perda de imunobiológicos por validade são ressaltadas:

“*Quando a gente recebe, a gente coloca as vacinas que já estão na geladeira para frente de acordo com a validade* (...) *a gente já chega elas para frente, pra gente ir utilizando as que já estavam aqui na unidade*” [ENF5-Nordeste].

A variação na frequência da supervisão das salas de vacinação, seja realizada pelo enfermeiro da unidade, pelo RTIR ou pelo RTIM, pode influenciar significativamente a eficiência e a qualidade do processo de vacinação.

“*Da unidade de saúde não existe* (...) *não tem essa rotina da enfermeira fazer essa vistoria. O que acontece é a enfermeira da instância municipal, ela passa todo ano, faz essa visita na sala de vacinação, vê todos os itens se estão dentro do padrão* (...) *Também a enfermeira da instância regional, ela faz todo ano uma visita nas salas* (...) *A gente tem um apoio muito grande, tanto da instância municipal, quanto da instância regional, ou por grupo de aplicativo de comunicação, ou ligando, ou pessoalmente*” [TEC1-Sul].

A rotatividade de profissionais, devido a contratos temporários ou vínculos empregatícios atrelados ao período de gestão municipal, é um grande desafio para a continuidade do trabalho nas salas.

“*É a realidade de todos os municípios a questão de cargos comissionados ou contratados. Então existe essa mudança de quatro anos ou de oito anos* [período eleitoral], *então a gente perde aquele profissional que tem experiência*” [RTIM4-Nordeste].

Com a rotatividade frequente de profissionais, a experiência e o conhecimento adquiridos ao longo do tempo são perdidos e é necessário fornecer treinamento constante. Entretanto, a realização de capacitações nas salas ocorre de forma esporádica, com atenção às atualizações pontuais e com a participação de apenas parte da equipe:

“*Com a mudança de uma gestão municipal, se não tiver sempre educação permanente, acaba perdendo o acesso com os profissionais, tendo que capacitar novos profissionais* (...) *e por conta disso muitas das vezes nós temos alguns desvios de fluxo*” [RTIE3-Nordeste].

“*Toda vez que tem alguma mudança ou alguma atualização eles convocam os técnicos de enfermagem, juntamente com a enfermeira. Nem sempre vai todo mundo junto, porque a unidade não pode ficar vazia*” [ENF6-Sudeste].

O *joint-displays* apresenta a combinação dos dados quantitativos e qualitativos ao estabelecer as conexões que evidenciaram as metainferências, que englobaram diversos aspectos como: o empenho na conservação de imunobiológicos; a fragilidades nas estruturas físicas e nos processos de trabalho nas salas de vacinação; ao impasses decorrentes da inexperiência de gestores municipais em relação à vacinação; a diversidade climática entre as regiões brasileiras; e a incompatibilidade das normativas estabelecidas pelo PNI (Quadro 1).

**Quadro 1:** Conexão dos dados quantitativos e categorias temáticas às metainferências, Brasil, 2023.

RESULTADOS QUAN → qual	RESULTADOS qual	METAINFERÊNCIAS
SEMPRE [n (%)]	CATEGORIAS TEMÁTICAS	
Uso de caixas térmicas de poliuretano (n = 218; 77,8%) Sala de vacinação exclusiva para vacinação (n = 223; 79,6%) Profissional capacitado (n = 136; 48,6%) Incidência de luz solar/calor nos equipamentos (n = 16; 5,8%) Substituição das bobinas de gelo reutilizáveis vencidas (n = 163; 58,2%) Manutenção preventiva (n = 143; 51,0%) Recebimento de informações sobre atualizações (n = 197; 70,4%) Disponibilidade de baterias ou geradores (n = 77; 27,6%) Presença de outros instrumentos de medição de temperatura (n = 74; 26,5%) Sala equipada com câmara refrigerada (n = 168; 60,0%) Transporte em carro climatizado (n = 162; 57,8%) Sala de vacinação climatizada (n = 189; 67,5%)	Estrutura das salas de vacinação: realidades da pluralidade brasileira O processo da manutenção da cadeia de frio: o cotidiano das salas de vacinação	As evidências reforçam um empenho pela manutenção da cadeia de frio nas salas de vacinação, contudo, demonstram fragilidades associadas à estrutura física e a processos de trabalhos que se entrelaçam às suas realidades, gerando repercussões negativas nesta cadeia A atuação de profissionais, em cargos de gestão municipal, sem conhecimentos técnicos acerca da cadeia de frio leva a impasses que podem repercutir na efetividade do processo de vacinação e interrupções do serviço A diversidade climática brasileira exige salas climatizadas para manter os imunobiológicos nas temperaturas recomendadas O uso de refrigerador doméstico ainda é uma realidade de muitas salas de vacinação brasileiras, situação em desacordo com as regulamentações propostas pelo PNI A disponibilidade de baterias ou geradores, assim como a manutenção preventiva são elementos cruciais para a conservação de imunobiológicos, sendo evidenciado como itens minimizado nas salas de vacinação A rotatividade de profissionais fragiliza os processos de trabalho, repercutindo no cotidiano das salas de vacinação, especialmente no que tange às ações de educação permanente e a continuidade da assistência

PNI: Programa Nacional de Imunizações.

## Discussão

Os resultados apontam para o empenho dos profissionais atuantes em todas as instâncias da cadeia de frio em garantir a manutenção da conservação dos imunobiológicos como uma potencialidade. Em contrapartida, foram identificadas fragilidades relacionadas a questões estruturais, como particularidades das condições geográficas entre as regiões, infraestrutura, insumos materiais e recursos tanto humanos quanto financeiros. Além disso, questões de processo, como organização e gestão do trabalho, rotatividade e capacitação profissional, também foram destacadas como áreas de vulnerabilidade.

Foram evidenciadas desigualdades inter-regionais com maior ênfase nas salas de vacinação da Região Nordeste, refletindo disparidades já conhecidas acerca de investimento e recursos materiais e humanos entre as regiões brasileiras ^15^. Embora as normativas estabelecidas pelo PNI se refiram a todas as salas do contexto nacional, a grande extensão territorial brasileira somada às condições socioeconômicas e demográficas repercutem na organização desses serviços nas distintas regiões ^16^. A economia nacional se concentra nas regiões Sul e Sudeste, enquanto as regiões Norte e Nordeste apresentam menor desenvolvimento socioeconômico ^17^ ^,^ ^18^. Esse cenário pode impactar as condições operacionais para conservação de imunobiológicos nas salas devido aos impasses relacionados à disponibilidade financeira.

Estudos evidenciam desafios e áreas que precisam ser melhoradas no contexto das condições das salas de vacinação brasileiras, corroborando os achados deste estudo. Em São Luís, no Maranhão, um estudo evidenciou que 89,6% das salas não estavam adequadas, enquanto o restante se configuraram como críticas, não atendendo às normas do PNI ^19^. E um estudo realizado em Fortaleza demonstrou uso de refrigeradores em estado prejudicado, com a limpeza inadequada, temperaturas inapropriadas e manutenção preventiva ausente ^20^. Outro estudo, realizado em 25 salas de vacinação brasileiras e com dados oriundos do PlanificaSUS (https://planificasus.com.br/index.php) em 2019, demonstrou irregularidades no condicionamento e na logística da cadeia de frio, com maior ênfase para as regiões Norte e Nordeste ^15^.

Tornou-se evidente por este estudo que, embora a exclusividade da sala de vacinação seja uma normativa estipulada pela Anvisa ^21^, ainda há uma parcela (20,4%) dessas com uso conjunto para outras ações que não somente as relacionadas à vacinação. Um estudo realizado em Aracaju (Sergipe) (21,4%) ^22^ e um estudo com dados do Programa de Melhoria do Acesso e da Qualidade da Atenção Básica (PMAQ-AB) dos municípios brasileiros (27,3%) ^23^ corroboram tal conformação. Nos relatos qualitativos, foram mencionados o uso da sala para realização de consultas, administração de outros medicamentos, teste do pezinho, entre outras ocorrências.

Outra evidência levantada foi a limitada disponibilidade de baterias ou geradores, manutenção preventiva e planos de contingências, indicando a necessidade de reformulações nessa estrutura. É crucial ressaltar que, em um país com vastas extensões territoriais e diversas condições geográficas, esses itens são essenciais para assegurar a manutenção efetiva da cadeia de frio. O uso de equipamentos de armazenamento adequados deve ser atrelado a análises periódicas de seus desempenhos a fim de evitar futuros problemas ^19^ ^,^ ^24^. Além do mais, haja vista a possibilidade de instabilidade e falhas da rede elétrica, torna-se relevante dispor de fontes de energia reservas nas salas ^25^ ^,^ ^26^ em associação à planos de contingência efetivos e executáveis para situações críticas.

Falhas na conservação dos imunobiológicos podem levar a perdas desses produtos e, consequentemente, incorrer no aumento de custos aos PNI. Um estudo realizado no noroeste paulista evidenciou que 41,4% das doses vacinais perdidas se relacionavam a motivos estruturais das salas de vacinação ^27^. Outro estudo realizado na instância estadual da cadeia de frio do Ceará mostrou que o principal motivo de perdas físicas de imunobiológicos foi a queda de energia elétrica, relatando que tais adversidades ocorreram em finais de semana ou feriados, em que o tempo de exposição a temperaturas fora da faixa ideal ultrapassou os limites preconizados pelo PNI. Evidenciou-se também ausência de contrato de manutenção preventiva e corretiva nos municípios ^28^, que foi uma realidade observada também nas salas analisadas neste estudo.

Os resultados deste estudo no que tange ao uso de câmaras refrigeradas apontam para uma disparidade entre as recomendações e a realidade nas salas de vacinação, corroborando com as outras análises do contexto nacional e internacional ^20^ ^,^ ^29^ ^,^ ^30^. Apesar das diretrizes estabelecidas pela Anvisa, em 2017 ^21^, e dos incentivos financeiros disponibilizados pelo Ministério da Saúde para a aquisição de tais equipamentos ^31^, em 2019, ainda se constata a utilização de refrigeradores domésticos em parte (40%) das salas brasileiras. Tais achados necessitam de elucidação visando identificar as possíveis barreiras que impedem a adoção mais ampla das câmaras refrigeradas, mesmo diante de recomendações e incentivos.

Embora menos onerosos, os refrigeradores domésticos não são eficazes na manutenção das temperaturas ideais requeridas ^24^, devendo ser substituídos por câmaras refrigeradas, que possuem melhor capacidade de homogeneização e estabilidade térmica.

Outro aspecto destacado em parcela das salas (22,2%) foi a não utilização de caixas de poliuretano, que são recomendadas por sua resistência, durabilidade e facilidade de higienização ^1^. Em vez disso, devido à sua escassez ou precariedade, relatou-se o uso de caixas de poliestireno expandido (isopor), que geralmente são destinadas ao transporte de imunobiológicos entre as instâncias da cadeia de frio.

Dispositivos para monitoramento e controle da temperatura devem ser incorporados aos equipamentos de refrigeração, em especial nas caixas térmicas para transporte, a fim de propiciar uma mensuração precisa das temperaturas ^1^ ^,^ ^30^. Há uma infinidade de instrumentos para tal, como os que incluem sistemas de monitoramento acurado e automatizado, os *data loggers*. Mesmo recomendado pelo PNI brasileiro ^1^, o uso de tais ainda é pouco recorrente nas salas de vacinação, prevalecendo a utilização de termômetros de momento, máxima e mínima digitais em razão de seu custo financeiro.

Recomenda-se o distanciamento dos equipamentos da incidência de luz solar ou de outras fontes de calor, além do uso de aparelhos de arcondicionado nas salas, visando o controle da temperatura ambiental e a minimização de riscos decorrentes das exposições dos imunobiológicos a temperaturas desnecessárias ^1^. Vale destacar que a diversidade climática encontrada nas regiões brasileiras exige salas climatizadas para manter os imunobiológicos dentro das temperaturas recomendadas, situação encontrada em parte das salas avaliadas neste estudo (67,5%).

As evidências destacaram a falta de veículos apropriados, assim como impasses na requisição desses e condições não recomendadas para o transporte de imunobiológicos. O transporte representa uma prática de risco para a eficácia desses produtos, uma vez que frequentemente carece de monitoramento durante o procedimento ^32^ ^,^ ^33^. Outro aspecto a ser enfatizado é a distância e a acessibilidade entre as instâncias municipais e as salas de vacinação, especialmente em áreas rurais, nas quais obstáculos físicos e geográficos podem causar entraves ^24^ ^,^ ^26^. Tais fatores podem prejudicar o transporte e, por conseguinte, resultar em perdas de imunobiológicos e estagnação dos processos de vacinação.

Foram apontadas dificuldades para o armazenamento de imunobiológicos nas instâncias municipais e nas salas de vacinação. Com a introdução de novos imunobiológicos no PNI e a realização de campanhas, há uma crescente demanda que sobrecarrega o armazenamento nessas instâncias. Esse cenário é ainda mais desafiador nos municípios de pequeno porte. Geralmente, nessas localidades com poucas salas, uma delas é designada para funcionar como instância municipal. No entanto, nem sempre são equipadas de acordo com a necessidade de armazenamento, além de apresentarem espaço físico reduzido.

Fragilidades associadas a processos de trabalhos também impactam na manutenção da cadeia de frio. A ausência de gerenciamento, baixa capacidade técnica e supervisão insuficiente são fatores atrelados ao desconhecimento da relevância da manutenção e gestão dessa cadeia ^24^ ^,^ ^26^.

Embora se tenha observado que, em 72,5% das salas, o profissional enfermeiro realiza a vacinação, os dados qualitativos evidenciam um distanciamento desse profissional das ações de supervisão da cadeia de frio, frequentemente direcionadas e executadas pelos RTIM. A demanda excessiva de tarefas e a escassez de recursos humanos são desafios significativos que limitam a participação mais ativa desses profissionais nas atividades de vacinação ^34^.

Uma equipe de profissionais capacitada e supervisionada está entre os fatores condicionantes para boas práticas de manutenção da cadeia e oferta de imunobiológicos efetivos ^2^ ^,^ ^3^. O engajamento da equipe de enfermagem em prol dos processos que envolvem a conservação de imunobiológicos é de suma importância, abrangendo o conhecimento adquirido, as trocas de experiências, a busca e a veiculação de informações acerca das normas técnicas e também a sistematização e empenho nos processos de trabalho ^29^. No entanto, este estudo evidenciou que, embora 70,4% dos profissionais tenham relatado sempre receber informações sobre as atualizações em imunização, apenas 48,6% relataram sempre participar de capacitações para a devida atuação nas salas. Esse achado corrobora com estudo realizado em Fortaleza, onde 31,5% dos profissionais nunca haviam participado de nenhuma capacitação na área ^20^.

A ausência de capacitações periódicas e restritas à parte dos profissionais envolvidos nas ações de vacinação podem fomentar práticas inadequadas na execução de procedimentos, os quais podem ser desconhecidos e/ou negligenciados pela equipe ^30^ ^,^ ^34^. Tal condição é evidenciada pela realização da ambientação das bobinas de gelo reutilizáveis de forma distinta do recomendado, o que pode levar a riscos potenciais de exposição dos imunobiológicos a temperaturas negativas.

Nesse ínterim, a constante atividade de supervisão, associada a capacitações periódicas, faz com que o reconhecimento situacional e os conhecimentos adquiridos se transformem em práticas efetivas de gestão da cadeia de frio ^2^ ^,^ ^3^. Dessa forma, a EACI se mostrou de fácil aplicabilidade e caracterizada como um instrumento efetivo que pode auxiliar o processo de supervisão nas salas de vacinação brasileiras, proporcionando a identificação de fragilidades e norteando a implementação de intervenções para a melhoria na conservação dos imunobiológicos.

A rotatividade dos profissionais se configura como outra fragilidade que impacta no processo de conservação de imunobiológicos ^35^. Como evidenciado neste estudo, a precariedade e temporalidade dos contratos de trabalho leva à rotatividade, a qual incide diretamente nas ações de vacinação, comprometendo a qualidade da assistência, além de fazer-se necessária a implementação de capacitações recorrentes. Destaca-se ainda que esse fator impossibilita a criação de vínculos e a longitudinalidade das ações de vacinação.

No que tange às limitações deste estudo, o caráter autorrelatado da aplicação da EACI e a realização da entrevista de modo não presencial pode ter levado ao mascaramento de entraves e potencialidades da conservação de imunobiológicos das salas de vacinação. Outra limitação se refere ao uso de uma plataforma online para mediação das entrevistas da etapa qualitativa, uma vez que isso não permitiu adentrar com profundidade na realidade estudada, limitando a interação e a compreensão dos contextos específicos. Entretanto, ambas as técnicas possíveis, em razão da dimensão do cenário e das barreiras impostas pela pandemia de COVID-19, proporcionaram evidências relevantes para análise no nível nacional, das salas de vacinação brasileiras e no que tange a conservação de imunobiológicos.

## Conclusão

Os achados possibilitaram conhecer realidades e pluralidades da cadeia de frio nas distintas salas de vacinação distribuídas nas regiões brasileiras, permitindo a identificação de potencialidades e fragilidades na conservação de imunobiológicos relacionadas às estruturas e a processos de trabalho que requerem aprimoramento. Eles forneceram informações para o suporte nas tomadas de decisões baseadas nos reais contextos dessas salas.

Pautando-se nos entraves evidenciados, é sugerida a substituição dos refrigeradores domésticos por câmaras refrigeradas e a compra de ar-condicionado; a disponibilidade de baterias para falhas da rede elétrica; a organização da escala de trabalho para que haja profissionais de forma exclusiva nas salas de vacinação; a licitação para contratação de serviços de manutenção preventiva; e a sensibilização dos gestores para proposição de concursos públicos, a fim de se aprimorar a assistência ofertada. Não menos importante, também é preciso a designação de gestores com base na experiência e competência técnica, garantindo uma gestão mais eficaz e focada na melhoria contínua dos serviços de vacinação.

Salienta-se a necessária continuidade de estudos que analisem a conservação de imunobiológicos nas salas de vacinação a fim de certificar o adequado acondicionamento, manuseio e oferta efetiva deles à população.
