# Supplementary material for: Analysis of the operational conditions to preserve immunobiological products in vaccination rooms in Brazil: a mixed study
Source: Cad Saude Publica. 2024 Aug 23;40(7):e00014924. [Article in Portuguese] doi: 10.1590/0102-311XPT014924 (PMC11346612; doi:10.1590/0102-311XPT014924)
Supplement: Supplementary file 1 [file 1678-4464-csp-40-07-PT014924-s.pdf]

Material Suplementar

Roteiros semiestruturados norteadores para a etapa qualitativa da pesquisa: análise das condições operacionais para conservação de imunobiológicos nas salas de vacinação do Brasil: estudo misto.

**ROTEIRO NORTEADOR (responsável técnico em imunização estadual)**	
**1.**	Que fatores influenciam para a manutenção da cadeia de frio de imunobiológicos nos municípios?<br>• Quais as dificuldades<br>• Quais as potencialidades
**2.**	Como se dá o planejamento da logística da cadeia de frio no Estado?<br>• Há regionais/distritos<br>• Insumos<br>• Instrumentos para monitoramento das temperaturas (mapas/planilhas)
**3.**	Como é realizado o transporte dos imunobiológicos da instância estadual para os municípios?<br>• Como são realizadas as entregas<br>• Instrumentos de medições<br>• Meios de transporte<br>• Os municípios buscam ou recebem<br>• Quem acompanha o trajeto
**4.**	Como se dá o financiamento para equipamentos e insumos da cadeia de frio no Estado?<br>• Subfinanciamento ou não<br>• Há prioridades nas compras
**5.**	Deseja fazer algum comentário adicional?<br>• Neste espaço você pode relatar algo acerca de alguma atividade/processo realizada e ou alguma peculiaridade relativa a sala de imunização que você atua, e que não esteja contemplado no questionário. Caso não tenha nada, favor deixar o campo em branco.
**ROTEIRO NORTEADOR (responsável técnico em imunização regional)**	
**1.**	Que fatores influenciam para a manutenção da cadeia de frio de imunobiológicos na sua regional/distrito?<br>• Quais a dificuldades<br>• Quais a potencialidades
**2.**	Como se dá a logística da cadeia de frio na regional/distrito?<br>• Como é realizado o transporte do nível regional para o nível municipal.<br>• Há uma central de imunização regional<br>• Insumos (caixas térmicas; Termômetros, registradores eletrônicos, mapas/planilhas)<br>• Equipamentos (Câmaras refrigeradas, Câmara fria)<br>• Como são realizadas as entregas<br>• O município busca ou recebe<br>• Meios de transporte<br>• Quem acompanha o trajeto<br>• A caixa é monitorada com termômetros<br>• Há controle da temperatura no momento da entrega nos municípios
**3.**	Como se dá a aquisição de equipamentos e insumos para a cadeia de frio da regional/distrito?<br>• Subfinanciamento ou não<br>• Há prioridades nas compras
**4.**	Como é o processo de contratação e capacitação de profissionais para atuação na regional/distrito?<br>• Os profissionais passam por capacitação antes da atuação<br>• São supervisionados por um profissional durante um período
**5.**	Como se dá o plano de contingência para casos de falta de energia elétrica na regional/distrito??<br>• Quem é comunicado<br>• Para onde são levados os imunobiológicos
**6.**	Quando os imunobiológicos são colocados sob suspeita na regional/distrito?<br>• Como ocorre este processo<br>• Os profissionais das salas sabem sobre os requisitos<br>• Quem é comunicado<br>• Os imunobiológicos são realocados
**7.**	Como ocorre a manutenção da geladeira/câmara refrigerada na regional/distrito?<br>• Há uma empresa ou profissional para tal atividade<br>• Qual a periodicidade<br>• Teste da bateria
**8.**	Como ocorre a supervisão das atividades realizadas na regional/distrito?<br>• Qual a periodicidade

	• Utiliza algum instrumento para tal atividade
**9.**	Deseja fazer algum comentário adicional?<br>• Neste espaço você pode relatar algo acerca de alguma atividade/processo realizada e ou alguma peculiaridade relativa a sala de imunização que você atua, e que não esteja contemplado no questionário. Caso não tenha nada, favor deixar o campo em branco.
**ROTEIRO NORTEADOR (responsável técnico em imunização municipal)**	
**1.**	Que fatores influenciam para a manutenção da cadeia de frio de imunobiológicos no seu município?<br>• Quais a dificuldades<br>• Quais a potencialidades
**2.**	Como se dá a logística da cadeia de frio no município?<br>• Como é realizado o transporte do nível regional para o nível municipal.<br>• Há uma central de imunização municipal<br>• Insumos (caixas térmicas; Termômetros, registradores eletrônicos, mapas/planilhas)<br>• Equipamentos (Câmaras refrigeradas, Câmara fria)<br>• Como são realizadas as entregas para as salas<br>• O município busca ou recebe (Estado/Regional/Distrito)<br>• Meios de transporte<br>• Quem acompanha o trajeto<br>• A caixa é monitorada com termômetros<br>• Há controle da temperatura no momento da entrega nas unidades de saúde
**3.**	Como é realizado o transporte dos imunobiológicos da instância municipal (central de vacinas) para as salas de imunização?<br>• Como são realizadas as entregas<br>• Meios de transporte<br>• As salas de imunização buscam ou recebem<br>• Quem acompanha o trajeto/rotatividade/profissional exclusivo<br>• A caixa é monitorada com termômetros<br>• Há controle da temperatura no momento da entrega nas unidades de saúde
**4.**	Qual a realidade das salas de imunização no município?<br>• Estrutura físicas das unidades de APS/Salas (casas adaptadas ou construídas/normas do PNI)
**5.**	Como se dá a aquisição de equipamentos e insumos para a cadeia de frio?<br>• Subfinanciamento ou não<br>• Há prioridades nas compras
**6.**	Como é o processo de contratação e capacitação de profissionais para atuação nas salas de imunização?<br>• Os profissionais passam por capacitação antes da atuação<br>• São supervisionados por um profissional durante um período
**7.**	Como se dá o plano de contingência para casos de falta de energia elétrica?<br>• Quem é comunicado<br>• Para onde são levados os imunobiológicos
**8.**	Quando os imunobiológicos são colocados sob suspeita?<br>• Como ocorre este processo<br>• Os profissionais das salas sabem sobre os requisitos<br>• Quem é comunicado<br>• Os imunobiológicos são realocados
**9.**	Como ocorre a manutenção da geladeira/câmara refrigerada das salas do município?<br>• Há uma empresa ou profissional para tal atividade<br>• Qual a periodicidade<br>• Teste da bateria
**10.**	Como ocorre a supervisão das atividades realizadas nas salas de imunização do município?<br>• Qual a periodicidade<br>• Utiliza algum instrumento para tal atividade
**11.**	Como ocorrem as capacitações acerca da temática vacinação?<br>• Qual a periodicidade<br>• Quais assuntos são abordados<br>• De qual maneira (palestras, convidados)
**12.**	No último ano houve alguma perda de imunobiológicos no município por falhas de conservação?<br>• Quais os motivos<br>• Falha no transporte (qual instância)
**13.**	Deseja fazer algum comentário adicional?<br>• Neste espaço você pode relatar algo acerca de alguma atividade/processo realizada e ou alguma peculiaridade relativa a sala de imunização que você atua, e que não esteja contemplado no questionário. Caso não tenha nada, favor deixar o campo em branco.
**ROTEIRO NORTEADOR (profissionais da equipe de enfermagem)**	
**1.**	Como você compreende a conservação dos imunobiológicos na sua rotina de trabalho?
**2.**	Como é a conservação dos imunobiológicos na sua Unidade de Saúde?

**3.**	Qual é a estrutura da sua sala de imunização?<br>• Estrutura físicas das unidades de APS/Salas (casas adaptadas ou construídas conforme normas do PNI)
**4.**	Como é realizado o transporte dos imunobiológicos da instância municipal (central de vacinas) para as salas de imunização?<br>• Como são realizadas as entregas<br>• Meios de transporte<br>• Os municípios buscam ou recebem<br>• Quem acompanha o trajeto<br>• Periodicidade da requisição (semanalmente? quinzenal?)
**5.**	Como é realizado o procedimento de montagem das caixas térmicas para o armazenamento dos imunobiológicos, no dia-a-dia?<br>• Que materiais são utilizados<br>• Ambientação das bobinas<br>• Imunobiológicos em recipientes (evitar conta direto com as bobinas)<br>• Limpeza da caixa térmica para uso diário<br>• Condições da caixa térmica (isopor, Poliuretano)
**6.**	Como se dá a organização dos imunobiológicos na geladeira/câmara refrigerada? E nas caixas térmicas?<br>• Sistema PEPS<br>• Divisão (vacinas bacterianas e virais)<br>• Organização (adulto/criança/identificação)
**7.**	Durante o período de trabalho, como ocorre o monitoramento das temperaturas da geladeira/câmara refrigerada e das caixas térmicas?<br>• Registro dos mapas de temperatura (periodicidade)
**8.**	Como se dá o plano de contingência para casos de falta de energia elétrica?<br>• Como é o procedimento (suspensão das atividades?)<br>• Quem é comunicado<br>• Para onde são levados os imunobiológicos
**9.**	Quando os imunobiológicos são colocados sob suspeita?<br>• Conhecimento sobre o tema<br>• Como ocorre este processo<br>• Quem é comunicado<br>• Os imunobiológicos são realocados
**10.**	Como é realizada a limpeza da geladeira/câmara refrigerada e das caixas térmicas?<br>• Remanejamento dos imunobiológicos<br>• Quem realiza<br>• Qual periodicidade<br>• Os imunobiológicos são realocados
**11.**	Como ocorre a manutenção preventiva da geladeira/câmara refrigerada?<br>• Há uma empresa ou profissional para tal atividade<br>• Qual a periodicidade<br>• Teste da bateria
**12.**	Como ocorre a supervisão das atividades realizadas na sala de imunização?<br>• Enfermeiro da unidades de APS e/ou RTIM<br>• Qual a periodicidade<br>• Utiliza algum instrumento para tal atividade
**13.**	Como ocorrem as capacitações acerca da temática vacinação?<br>• Qual a periodicidade (último ano/atual houve alguma)<br>• Quais assuntos são abordados<br>• De qual maneira (palestras, convidados)<br>• Capacitações na unidade? Ou na central?
**14.**	No último ano houve alguma perda de imunobiológicos na sala de imunização por falhas de conservação?<br>• Quais os motivos<br>• Falha no transporte (qual instância)
**15.**	Deseja fazer algum comentário adicional?<br>• Neste espaço você pode relatar algo acerca de alguma atividade/processo realizada e ou alguma peculiaridade relativa a sala de imunização que você atua, e que não esteja contemplado no questionário. Caso não tenha nada, favor deixar o campo em branco.

APS: atenção primária à saúde; PNI: Programa Nacional de Imunização; RTIM: responsável técnico em imunização municipal.
